# Non-Invasive Determination of Cardiac Output in Pre-Capillary Pulmonary Hypertension

**DOI:** 10.1371/journal.pone.0134221

**Published:** 2015-07-30

**Authors:** Frédéric Lador, Philippe Hervé, Aurélien Bringard, Sven Günther, Gilles Garcia, Laurent Savale, Guido Ferretti, Paola M. Soccal, Denis Chemla, Marc Humbert, Gérald Simonneau, Olivier Sitbon

**Affiliations:** 1 Service de Pneumologie, Programme Hypertension Pulmonaire, Hôpitaux Universitaires de Genève, Genève, Switzerland; 2 AP-HP, Centre de Référence de l’Hypertension Pulmonaire Sévère, Département Hospitalo-Universitaire (DHU) Thorax Innovation (TORINO), Service de Pneumologie, Hôpital de Bicêtre, Le Kremlin Bicêtre, France; 3 Centre Chirurgical Marie-Lannelongue, Le Plessis-Robinson, France; 4 Département des Neuroscience Fondamentales, Centre Médical Universitaire, Université de Genève, Genève, Switzerland; 5 Université Paris–Sud, Faculté de Médecine, Le Kremlin Bicêtre, France; 6 Dipartimento di Scienze Cliniche e Sperimentali, Università di Brescia, Brescia, Italy; 7 Département de Physiologie, Université Paris-Sud, Faculté de Médecine-EA4533-APHP, Le Kremlin Bicêtre, France; Vanderbilt University Medical Center, UNITED STATES

## Abstract

**Background:**

Cardiac output (CO) is a major diagnostic and prognostic factor in pre-capillary pulmonary hypertension (PH). Reference methods for CO determination, like thermodilution (TD), require invasive procedures and allow only steady-state measurements. The Modelflow (MF) method is an appealing technique for this purpose as it allows non-invasive and beat-by-beat determination of CO.

**Methods:**

We aimed to compare CO values obtained simultaneously from non-invasive pulse wave analysis by MF (CO_MF_) and by TD (CO_TD_) to determine its precision and accuracy in pre-capillary PH. The study was performed on 50 patients with pulmonary arterial hypertension (PAH) or chronic thrombo-embolic PH (CTEPH). CO was determined at rest in all patients (n = 50) and during nitric oxide vasoreactivity test, fluid challenge or exercise (n = 48).

**Results:**

Baseline CO_MF_ and CO_TD_ were 6.18 ± 1.95 and 5.46 ± 1.95 L·min^-1^, respectively. Accuracy and precision were 0.72 and 1.04 L·min^-1^, respectively. Limits of agreement (LoA) ranged from -1.32 to 2.76 L·min^-1^. Percentage error (PE) was ±35.7%. Overall sensitivity and specificity of CO_MF_ for directional change were 95.2% and 82.4%, (n = 48) and 93.3% and 100% for directional changes during exercise (n = 16), respectively. After application of a correction factor (1.17 ± 0.25), neither proportional nor fixed bias was found for subsequent CO determination (n = 48). Accuracy was -0.03 L·min−1 and precision 0.61 L·min^−1^. LoA ranged from -1.23 to 1.17 L·min^−1^ and PE was ±19.8%.

**Conclusions:**

After correction against a reference method, MF is precise and accurate enough to determine absolute values and beat-by-beat relative changes of CO in pre-capillary PH.

## Introduction

Pre-capillary pulmonary hypertension (PH) is a haemodynamic condition that may lead to right heart failure, and that is defined by an increased resting pulmonary artery mean pressure (mPAP) due to elevated pulmonary vascular resistance (PVR) [1]. Consequently, cardiac output (CO), is a key diagnostic parameter and a major prognostic factor in diseases like pulmonary arterial hypertension (PAH) and chronic thromboembolic pulmonary hypertension (CTEPH) [1, 2], both characterized by such a condition.

Classical methods for CO measurement require right heart catheterisation (RHC) where the reference method is the direct Fick, although thermodilution (TD) or indirect Fick methods are widely preferred because of their relative simplicity in the clinical setting [1]. Hence, the availability of new, simple, reliable and non-invasive method for CO determination at rest and for CO changes in response to pharmacological interventions or during metabolic and volemic changes is desirable.

A promising technique for this purpose called Modelflow (MF) [3], relies on arterial pulse pressure wave analysis. Once corrected against a reference method, MF was shown to be a reliable and accurate procedure in healthy humans [4], requiring only the application of a finger plethysmographic cuff to the patient for the continuous monitoring of pulse pressure profiles, thus being simpler than any other method proposed so far.

To the best of our knowledge, the accuracy and precision of MF was never assessed in pre-capillary PH. This study aims to evaluate MF in PAH and CTEPH patients, by comparing CO values obtained by MF (CO_MF_) with values simultaneously determined on the same patients by TD (CO_TD_) during RHC procedures.

## Materials and Methods

### Study population

**Fig 1:**
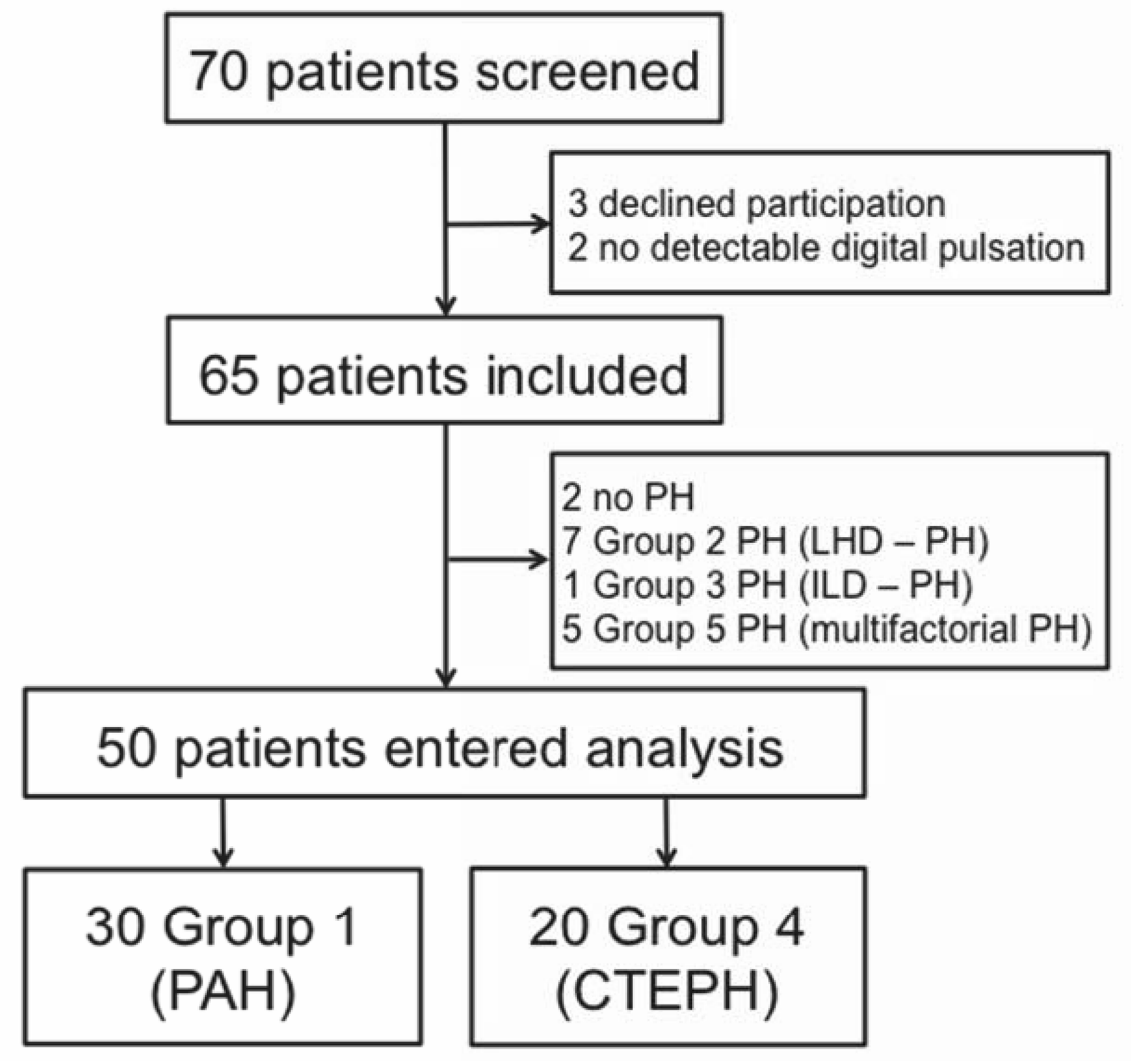
Study profile. PH: Pulmonary hypertension; LHD–PH: PH due to left heart disease; ILD–PH: PH due to interstitial lung disease; PAH: Pulmonary arterial hypertension; CTEPH: Chronic thromboembolic PH.

Seventy consecutive patients from the outpatient clinic and who underwent RHC for suspected or diagnosed pre-capillary PH within their routine workup were invited to participate (Fig 1). Patients with cardiac shunts were excluded, TD being potentially inaccurate in this condition. Patients with PH due to left heart disease (diagnostic Group 2 according to WHO classification, post-capillary PH) or PH with unclear or multifactorial mechanisms (diagnostic Group 5) were also excluded. In order to assess a homogeneous population, patients with PH due to lung disease were excluded because the physiopathology of pre-capillary PH is different in this group (diagnostic Group 3). 2 patients were also excluded from the study due to poor or no fingertip pulse pressure signal due to systemic sclerosis. Screening was not proposed to patients hospitalized in intensive care unit with acute right heart failure, haemodynamic shock or other life threatening condition. Analyses was finally performed on fifty patients with PAH (diagnostic Group 1, n = 30) or CTEPH (diagnostic Group 4, n = 20). This study was approved by a local ethics committee (comité de protection des personnes, Ile de France VIII, Hôpital Ambroise Paré, Boulogne-Billancourt, France) and was performed in the French reference centre for severe PH in Paris. All patients gave written informed consent.

### Right heart catheterisation

Haemodynamic evaluation was performed in a supine position. Electrocardiogram and arterial oxygen saturation (pulse oximetry) were monitored continuously. Mean systemic arterial pressure (MAP) was measured at the brachial artery (Dynamap 1800; Critikon, Tampa, FL, USA). RHC was performed using the modified Seldinger technique with an 8F sheath inserted in the jugular, basilic or cephalic vein. The Swan-Ganz catheter was a 7F, two-lumen, TD and pressure-measuring tipped catheter (Corodyn TD; Braun Medical, Bethlehem, PA, USA). The zero-level reference was determined at mid-thoracic line. Resting haemodynamic evaluation included measures of right atrial pressure (RAP), mPAP, pulmonary artery wedge pressure (PAWP), CO_TD_. PVR was calculated as (mPAP–PAWP)/ CO_TD_, and systemic vascular resistance as (MAP–RAP)/ CO_TD_. Cardiac index was calculated as CO divided by body surface area. Fluid challenge was performed by infusion of 500 ml of isotonic saline solution in five minutes, and haemodynamic parameters reassessed afterward. Vasoreactivity test was performed with inhaled nitric oxide according to current recommendations [1] prior to haemodynamic reassessment.

### Cardiac output by thermodilution

In each patient, the positioning of the Swan-Ganz probe was confirmed by fluoroscopic control. CO_TD_ was determined by injection of 10 ml of iced-cold sterile, isotonic glucosaline solution through the proximal catheter’s lumen. The time course of temperature changes was recorded by the thermistor at the distal end of the probe. Three consecutive bolus injections were performed for each condition. The mean value of the three measurements was calculated if the difference between highest and lowest values was ≤ 10%. Otherwise, two more measurements were performed, the highest and lowest values were deleted, and the mean value of the three remaining measurements was calculated.

### Cardiac output by Modelflow

CO_MF_ was determined from continuous non-invasive recording of arterial pulse pressure profiles by a Portapres system (TNO-TPD, Amsterdam, The Netherlands). The photoplethysmographic cuff of Portapres was positioned on the index or middle finger contralateral to the vascular lines. The zero-level reference was placed according to the manufacturer’s instructions. From pulse pressure profiles, beat-by-beat heart rate (HR) and stroke volume (SV) were calculated with the MF model, using the Beatscope 1.1a software (TNO-TPD, Amsterdam, The Netherlands) developed for this purpose.

The MF method for the beat-by-beat assessment of CO makes it possible to reconstruct aortic blood flow by simulating a three-element non-linear and time varying model of aortic compliance [3]. Numerical integration of flow during systole then yields SV. CO_MF_ is finally computed as SV times the corresponding HR.

The CO_MF_ value in each condition was the mean value calculated over 100 consecutive beats, starting simultaneously to the first of each series of CO_TD_ procedure. Individual correction factors were calculated with the resting (basal) values as described elsewhere [4] and used to recalculate CO_MF_ (CO_MFcorr_.) in other conditions.

### Exercise haemodynamics

Exercise haemodynamic evaluation was performed supine on an electromagnetically-braked cycle ergometer (Cycline 100; Tecmachine, Andrezieux-Boutheon, France) secured to the catheterization table. Prior to exercise, 5 min of rest with feet installed on the bicycle (raised legs) were observed to ensure haemodynamic steady state after the expected increase of blood venous return to the heart (considered as “bicycle rest”). Then the patient pedalled at 60 rpm, the workload being increased stepwise by 20 W every 3 min from freeload cycling to a maximum of 60 W, depending on patient’s functional tolerance. CO was determined during the last minute of each exercise level. All measures were obtained at steady HR and pulmonary artery pressure.

## Statistics

Data are given as mean ± standard deviation (SD), unless otherwise stated. Data comparisons were performed by t-test for paired observations. Differences were considered significant when p < 0.05. Coefficient of variation was calculated as mean divided by SD for baseline CO values. Linear relationships were analysed by linear regression, accounting for the error on both X- and Yaxis. The York algorithm [5] implemented under Matlab (version 7.13.0.564, MathWorks, Natick, MA, USA) was used, allowing calculation of confidence intervals for intercept (*a* ± 1.96*SD) and slope (*b* ± 1.96*SD), and estimation of proportional bias (*a* significantly different from 1) and fixed bias (*b* significantly different from 0) between the two methods. Bland–Altman analyses [6] were performed to determine the degree of agreement between CO_MF_ and CO_TD_ and between CO_MFcorr._ and CO_TD_. The mean bias (accuracy), precision, 95% limits of agreements (LoA) and percentage error (PE) were calculated. Sensitivity and specificity of CO_MF_ for directional CO_TD_ increase or decrease in response to any procedure were determined. A directional change was considered false-negative when an increase of CO_TD_ ≥ 10% was not accompanied by a CO_MF_ increase ≥ 10%. A directional change was considered false-positive when a COTD increase < 10% or any CO_TD_ decrease was accompanied by an increase of CO_MF_ ≥ 10%.

## Results

**Fig 2:**
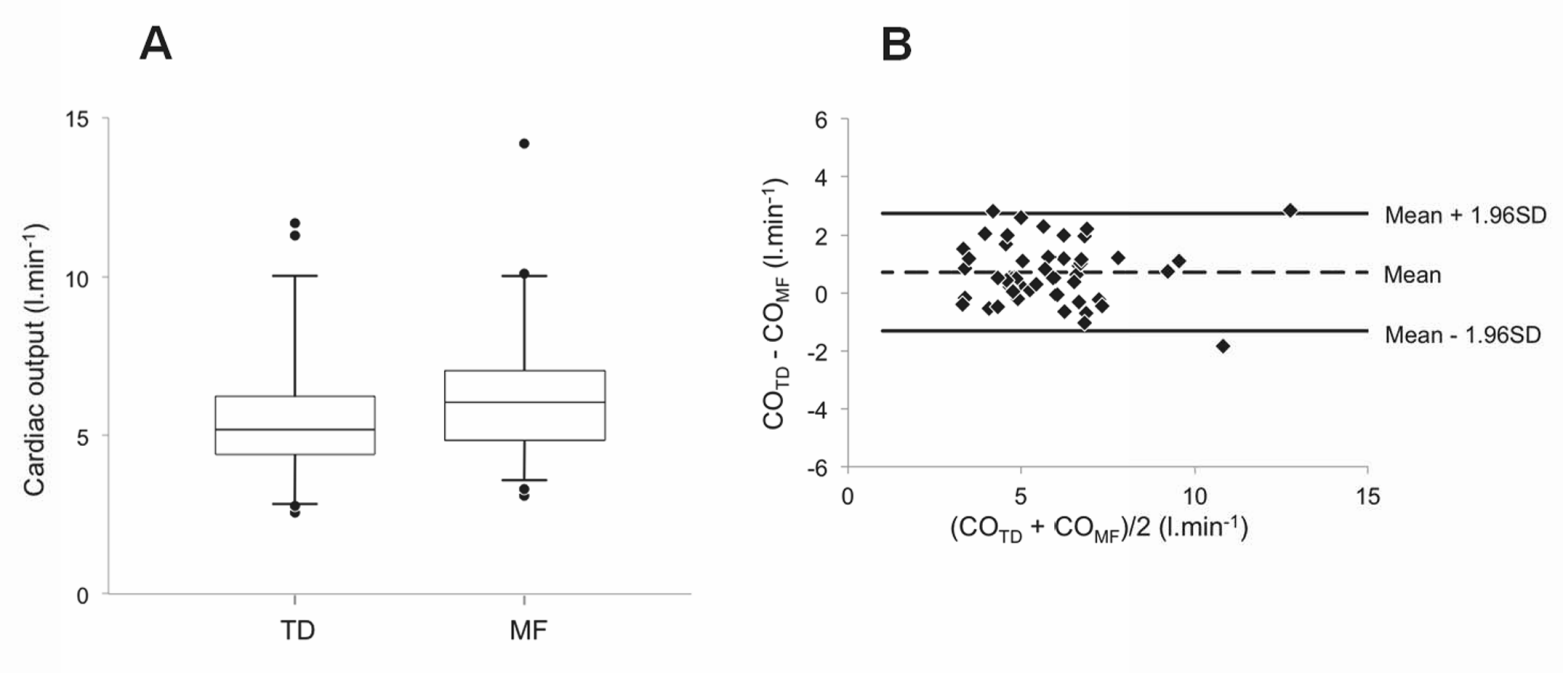
Comparison of baseline values of CO_MF_ and CO_TD_. Simultaneous determination of cardiac output by thermodilution (CO_TD_) and Modelflow (CO_MF_) in 50 patients with pre-capillary pulmonary hypertension. **(A)** The figure describes median (line), 25th to 75th percentile (box), 5th to 95th percentile (whiskers) and the dots represent outliers. The mean values for CO_TD_ and CO_MF_ were 5.46 ± 1.95 L·min^-1^ and 6.18 ± 1.95 L·min^-1^, respectively (p<0.05). **(B)** Difference between resting CO_MF_ and CO_TD_ values plotted against their mean. Broken line represents the mean (+ 0.72 L·min^-1^) and the solid lines the 95% limits of agreement (-1.32 to + 2.76 L·min^-1^).

**Fig 3:**
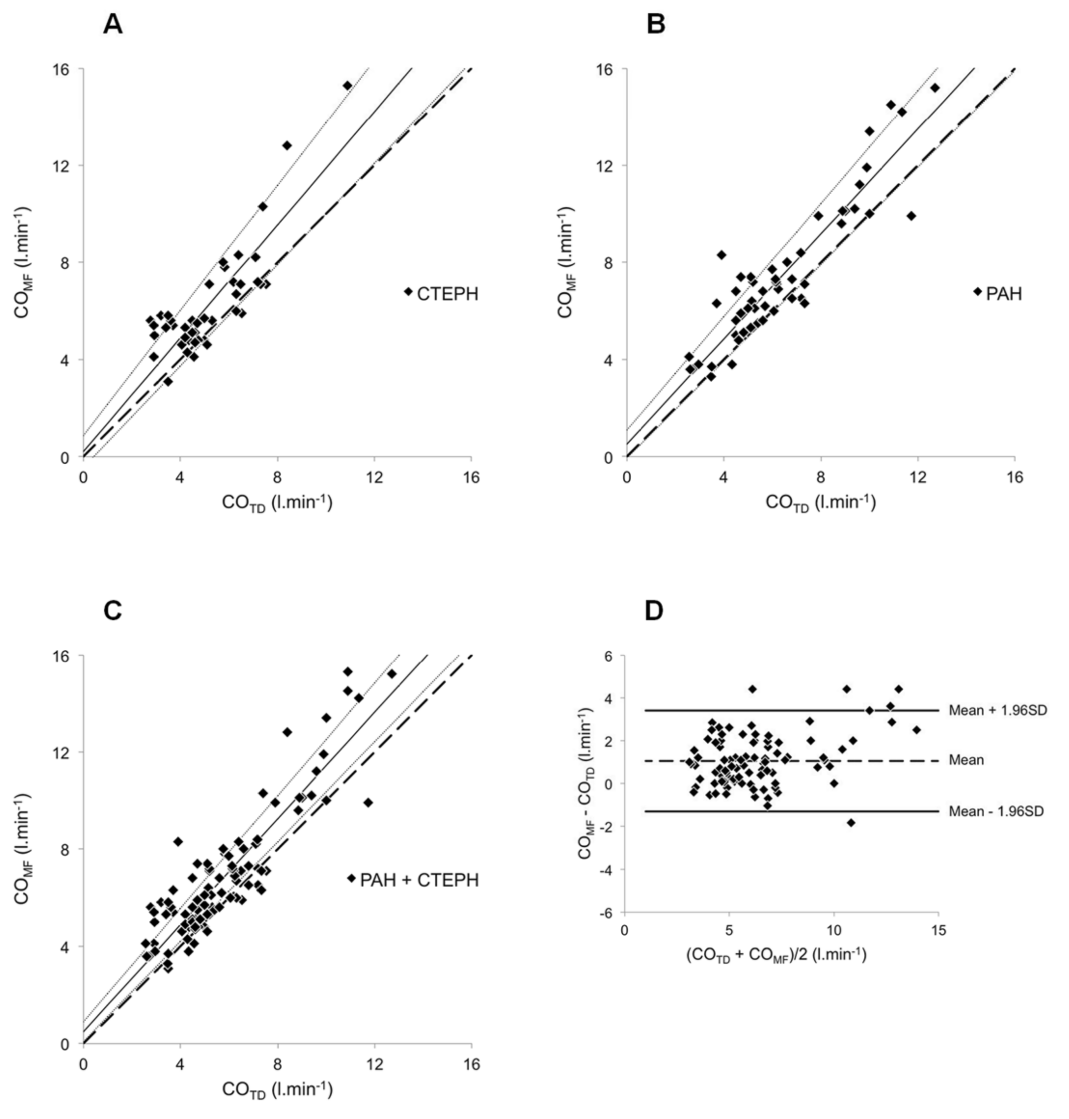
Relationship between CO_MF_ and CO_TD_. COMF determined in 50 patients (98 values) under various conditions (rest, fluid challenge, NO testing and exercise). CO_MF_ values were plotted against the corresponding CO_TD_ values for CTEPH patients **(A)** PAH patients **(B)** and all 50 patients **(C)**. **(D)** Difference between CO_MF_ and CO_TD_ values plotted against their mean. In (A), (B) and (C), the broken lines correspond to the lines of equality, solid lines are the mean regression lines and dotted lines delimit the confidence interval of the regression lines. In (D), broken line represents the mean (+ 1.05 L·min^-1^) and the solid lines the 95% limits of agreement (-1.30 to + 3.40 l.min^-1^).

**Fig 4:**
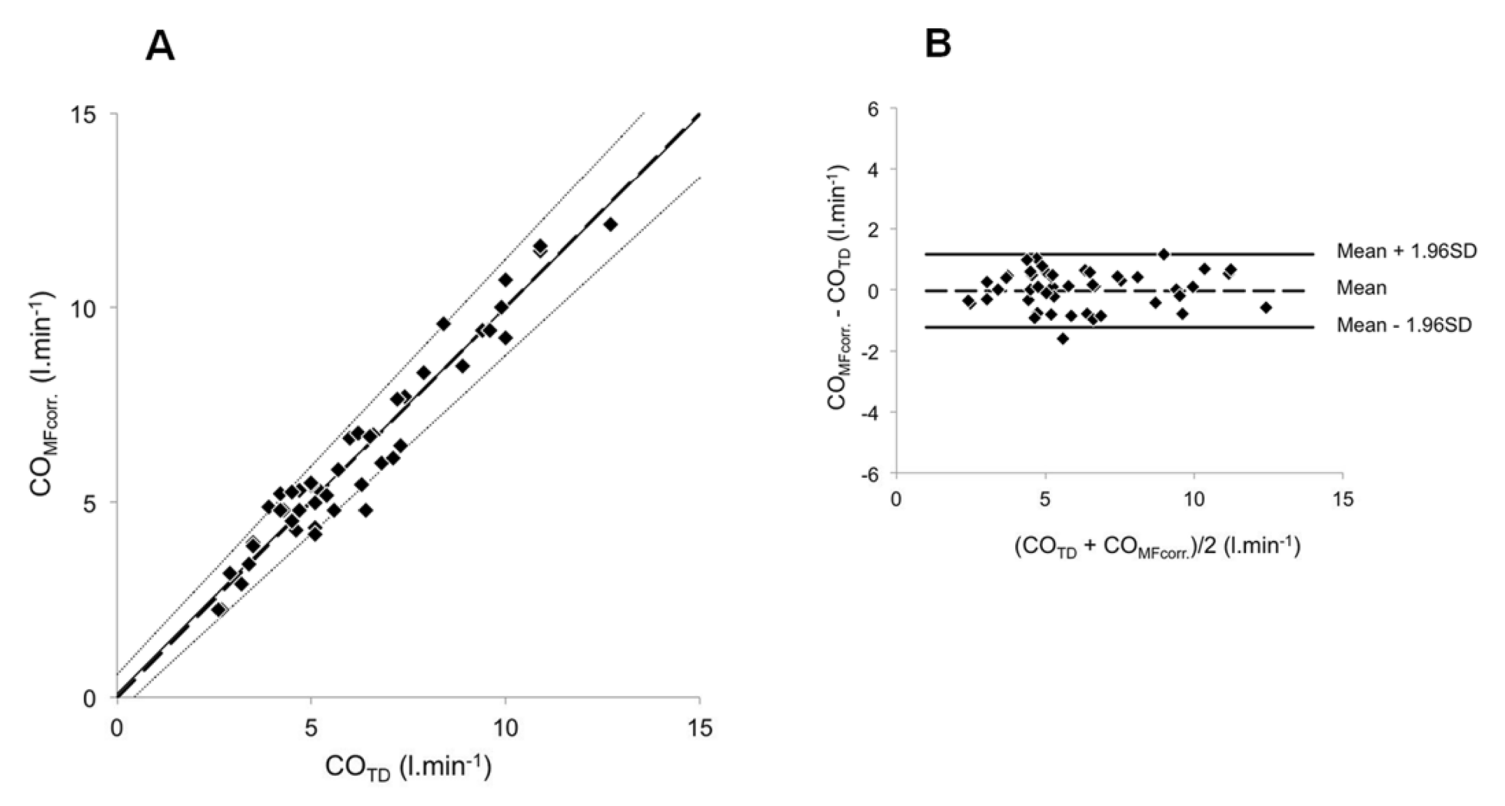
Relationship between CO_MFcorr._ and CO_TD_. COMFcorr. determined in 26 patients (48 values). **(A)** For each subject, CO_MFcorr._ values were plotted against the corresponding CO_TD_ values. The broken line corresponds to the line of equality, solid line is the mean regression lines and dotted lines delimit the confidence interval of the regression lines. **(B)** Difference between CO_MFcorr._ and CO_TD_ values plotted against their mean. Broken line represents the mean (-0.03 L·min^-1^) and the solid lines the 95% limits of agreement (-1.23 L·min^-1^ to +1.17 L·min^-1^).

**Fig 5:**
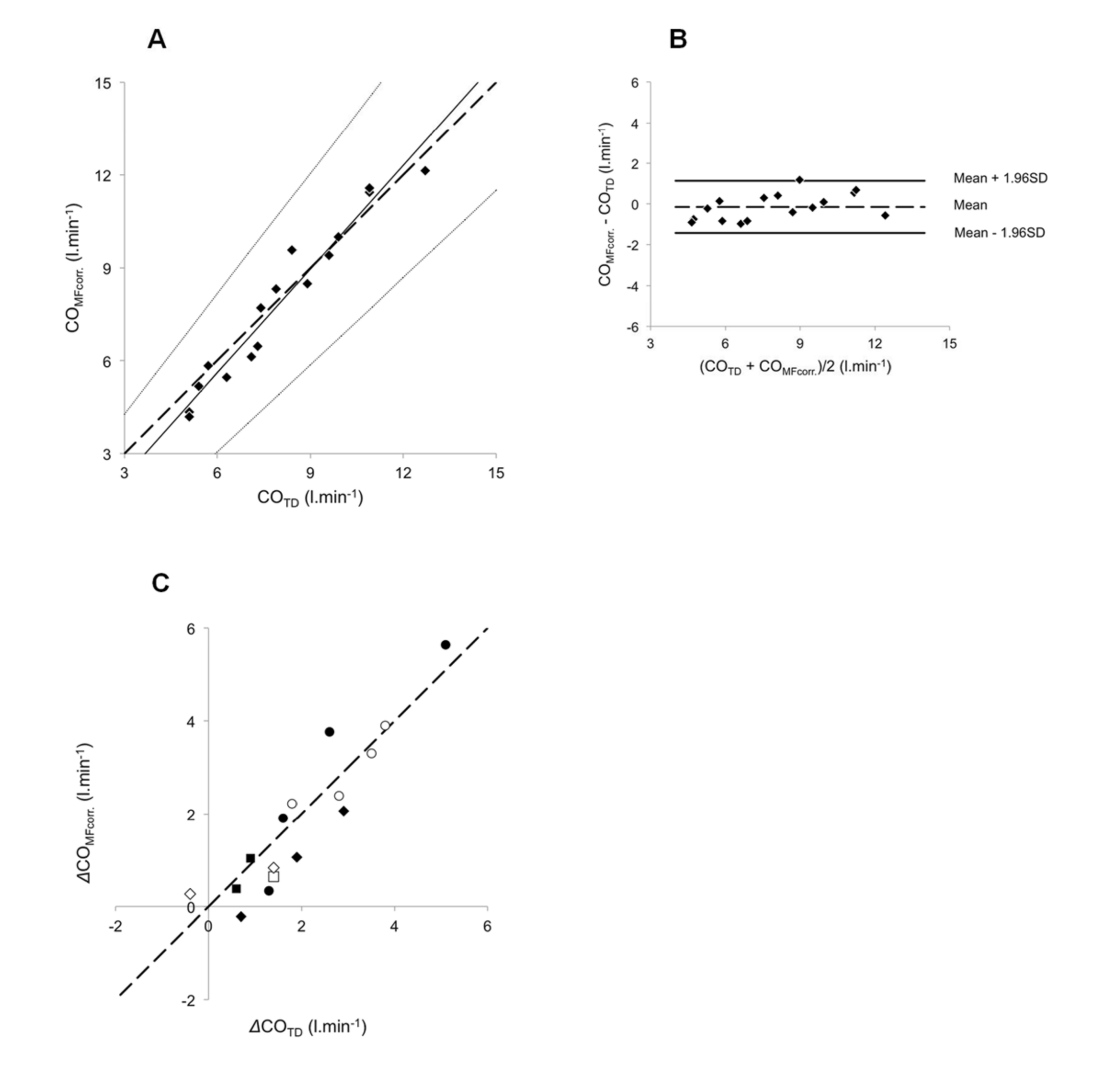
Relationship between CO_MFcorr._ and CO_TD_ during exercise. CO_MFcorr_ determined in 6 patients during exercise procedure. **(A)** For each subject, CO_MFcorr_ values were plotted against the corresponding CO_TD_ values. The broken line corresponds to the line of equality, solid line is the mean regression lines and dotted lines delimit the confidence interval of the regression lines. **(B)** Difference between CO_MFcorr_ and CO_TD_ values plotted against their mean. Broken line represents the mean (-0.15 L·min^-1^) and the solid lines the 95% limits of agreement (-1.42 l.min^-1^ to +1.12 L·min^-1^). **(C)** For each subject and workload, the increase (Δ) in CO_MFcorr_ from rest was plotted against the same corresponding CO_TD_ increase (ΔCO_TD_). The six different targets correspond to the six different patients. The broken line corresponds to the line of equality.

The clinical and haemodynamic data at baseline are shown in Tables 1, 2 and 3. The conditions and number of simultaneous CO_TD_ and CO_MF_ measurements are shown in Table 4. Baseline uncorrected CO_MF_ and CO_TD_ were 6.18 ± 1.95 and 5.46 ± 1.95 L·min^-1^, respectively, (p<0.05, Fig 2A). The coefficients of variation were 10.63% and 5.66% for CO_MF_ and CO_TD_, respectively (p<0.01). The Bland–Altman plot for baseline CO values appears in Fig 2B. Accuracy and precision were 0.72 and 1.04 L·min^-1^, respectively. LoA ranged from -1.32 to 2.76 L·min^-1^. PE was ±35.7%. The relationships between CO_MF_ and the corresponding CO_TD_ are reported in Fig 3A, 3B and 3C. For CTEPH (n = 43), PAH (n = 55) and overall (n = 98), the intercept *a* was 0.207 ± 0.336, 0.516 ± 0.294 and 0.493 ± 0.209, respectively. Corresponding slope *b* was 1.167 ± 0.062, 1.082 ± 0.043 and 1.095 ± 0.034 L·min^-1^, respectively. A proportional bias was found for CTEPH (Fig 3A). Merging groups implied both proportional and fixed bias (Fig 3C). The Bland–Altman plot for merged groups is shown in Fig 3D. The accuracy was 1.05 L·min^−1^ (p <0.01). The precision was 1.20 L·min^-1^. LoA ranged from -1.30 to 3.40 L·min^-1^. PE was ±37.9%. The overall sensitivity and specificity of CO_MF_ for directional change in response to any of the experimental manoeuvres (nitric oxide vasoreactivity test, fluid challenge or exercise) were 95.2% and 82.4%, respectively (n = 48). Mean calibration factor for CO_MF_ was 1.17 ± 0.25. CO_MFcorr._ in all conditions except baseline (which was used to determine the correction factor) are plotted against the corresponding CO_TD_ in Fig 4A. The regression line was characterized by *a* and *b* of 0.094 ± 0.254 L·min^−1^ and 0.990 ± 0.038, respectively. Neither proportional nor fixed bias was found. The Bland–Altman plot (Fig 4B) showed an accuracy of -0.03 l·min^−1^ (NS). Precision was 0.61 L·min^−1^. LoA ranged from -1.23 to 1.17 L·min^-1^. PE was ±19.8%. CO_MFcorr._ values at exercise (n = 16) are plotted against their corresponding CO_TD_ in Fig 5A. The regression equation had intercept *a* and slope *b* of -1.093 ± 0.760 and 1.117 ± 0.091 L·min^−1^, respectively. Neither proportional nor fixed bias was found. The Bland–Altman plot (Fig 5B) revealed a -0.15 L·min^−1^ accuracy (NS). Precision was 0.65 L·min^−1^. LoA ranged from -1.42 to 1.12 L·min^-1^. PE was ±16.4%. The changes of CO_MFcorr_ (ΔCO_MFcorr_) from baseline for each subject are plotted against the corresponding ΔCO_TD_ in Fig 5C. The sensitivity and specificity of CO_MF_ for directional change in response to exercise were 93.3%. and 100%, respectively (n = 16). In one case, a CO_MF_ decrease < 10% (-0.22 L·min^-1^) did not accompany a CO_TD_ increase ≥ 10% (+0.70 L·min^-1^) and was considered a false negative.

**Table 1:** Baseline characteristics of the study population.

		PAH (n = 30)	CTEPH (n = 20)	
			Not operated (n = 14)	Operated (n = 6)
Age	yrs	48.7 ± 15.6	64.3 ± 11.0	56.2 ± 19.8
Female	%	60.0	57.1	66.7
BMI	kg.m^-2^	25.0 ± 5.1	25.4 ± 4.9	31.5 ± 5.8
BSA	m^2^	1.77 ± 0.19	1.76 ± 0.23	1.89 ± 0.18
MAP	mmHg	90.9 ± 14.2	104.3 ± 20.4	95.0 ± 19.5
RAP	mmHg	5.8 ± 3.1	7.4 ± 5.2	4.3 ± 3.9
mPAP	mmHg	48.6 ± 14.9	43.0 ± 13.0	26.3 ± 7.6
PAWP	mmHg	9.2 ± 3.7	6.2 ± 2.4	9.3 ± 3.3
CO	L·min^-1^	6.0 ± 2.1	4.3 ± 1.3	5.5 ± 0.7
CI	L·min^-1^·m^-2^	3.4 ± 1.0	2.4 ± 0.5	2.9 ± 0.2
PVR	WU	7.5 ± 4.4	9.5 ± 4.5	3.2 ± 1.3
SVR	WU	15.9 ± 6.1	24.7 ± 10.1	17.1 ± 5.8

Data are presented as mean ± SD unless otherwise stated. BMI: Body mass index; BSA: Body surface area; MAP: Mean systemic arterial blood pressure; RAP: Right atrial pressure; mPAP: Pulmonary artery mean pressure; PAWP: pulmonary artery wedge pressure; CO: cardiac output, determined by thermodilution; CI: Cardiac Index; PVR: Pulmonary vascular resistance; SVR: Systemic vascular resistance; WU: Wood units.

**Table 2:** Diagnosis and treatment of the PAH population (%).

Subjects	30
WHO diagnostic subgroup	
Idiopathic	17 (56.7)
Heritable	2 (6.7)
Drugs and toxin induced	1 (3.3)
Associated^#^	9 (30.0)
Pulmonary veno-occlusive disease	1 (3.3)
Treatment naive (newly diagnosed^§^)	7 (23.3)
No specific drug therapy	1 (3.3)
PAH-specific drug therapy	22 (73.3)
ERA	16 (72.7)
PDE5i	12 (54.5)
Prostanoid	7 (31.8)
CCB	1 (4.5)
Monotherapy	11 (50.0)
Double combination therapy	7 (31.8)
Triple combination therapy	4 (18.2)

**Table 3:** Diagnosis and treatment of the CTEPH population (%).

Subjects	20
Newly diagnosed^§^	12 (60.0)
Inoperable**	2 (10.0)
Operated^¶^	6 (30.0)
PAH-specific drug therapy	5 (25.0)
ERA	5 (100)
PDE5i	4 (80.0)
Monotherapy	1 (20.0)
Double combination therapy	4 (80.0)

ERA: Endothelin receptors antagonists; PDE5i: phosphodiesterase-5 inhibitors; CCB: Calcium channel blockers. #Patients with PAH associated to connective tissue disease (n = 1), HIV infection (n = 2), Portal hypertension (n = 2) and Congenital heart disease after corrective cardiac surgery (n = 4).

§Patients diagnosed at the time of the present study.

**Due to distal lesions.

¶ Patients with persisting hemodynamic impairment at least 3 months after pulmonary endarterectomy (PEA).

**Table 4:** Conditions and number of simultaneous CO_TD_ and CO_MF_ measurements.

n	Rest (baseline)	NO testing	Fluid Challenge	Bicycle rest	Bicycle exercise	Total
PAH	30	7	4	5	9	**55**
CTEPH	20	9	1	6	7	**43**
**Total**	**50**	**16**	**5**	**11**	**16** ^#^	**98**

Repartition of the different single simultaneous CO_TD_ and CO_MF_ measurements. A total of 98 CO measurements were performed in the 50 patients. Each CO_TD_ is the mean of 3 TD measurements (see Methods). Each CO_MF_ is the mean of 100 consecutive beat-by-beat values (see Methods). 26 patients (PAH n = 12, CTEPH n = 14) had a total of 48 measurements in conditions other than basal (PAH n = 25, CTEPH n = 23). #6 patients performed incremental exercise (PAH n = 4, CTEPH n = 2) with the workload being increased stepwise by 20 W every 3 min to a maximal workload of 60 W depending of patient functional tolerance for a total of 16 CO determined during steady exercise (PAH n = 9, CTEPH n = 7). NO: Nitric oxide vasoreactivity test; Fluid Challenge: CO determined after infusion of 500 ml of isotonic saline solution in five minutes; Bicycle rest: CO determined after 5 min of rest with feet positioned on the pedals with raised legs.

## Discussion

The results of the present study show that MF applied to non-invasive pulse pressure profiles, once corrected against a reference method, offers a precise and accurate determination of CO in patients with PAH and CTEPH.

Uncorrected CO_MF_ overestimation of CO_TD_ was confirmed by the ranges of regression lines in Fig 3, displaced above the equality line when plotting CO_MF_ against CO_TD_ in CTEPH and merging groups. When applied to the PAH group only (Fig 3B), however, linear regression showed neither fixed nor proportional bias. These results are consistent with previous data, comparing MF applied to peripheral pulse pressure profiles to different reference methods in various clinical settings and populations [4, 7, 8] and confirm the need of a correction when determination of absolute CO values, more than relative changes, is needed. Conversely, when compared to TD at rest, non calibrated MF applied to pulse waves obtained from intra-arterial signal in cardiac surgery patients was reliable and superior to other CO monitoring devices, with acceptable LoA and PE [9]. These results were confirmed by a recent meta-analysis [10]. Another study compared CO_MF_ from arterial pulse pressure profiles recorded with Portapres and from intra-arterial radial catheter in healthy subjects [11]: non-invasive CO was significantly and systematically higher than CO from intra-arterial signal. The systematic difference was attributed to differences in pulse wave between radial and finger arteries. This may also partially explain the overestimation of CO_MF_ as compared to CO_TD_ in our study. Here, PE of CO_MF_ slightly exceeded the ±30% limit admitted for allowing interchangeability of a new technology with TD, as suggested by a meta-analysis of CO validation studies published by Critchley and Critchley [12]. Still, the LoA and PE values of our study were smaller than those obtained in PH patients for TD against direct Fick [13], thoracic bioimpedance against TD [14] and transthoracic bioreactance against TD or indirect Fick [15]. It is noteworthy that these two latter alternative methods for non-invasive CO determination are also promising, as discussed in a recent review that focused its performances in the assessment of fluid responsiveness using passive leg raising test and cardiac output response to exercise stress testing [16]. In our study, CO_MF_ was not compared with direct or indirect Fick: any assumption of a better correlation between MF and these methods would be speculative. Previously, determination of CO changes by acetylene rebreathing showed good correlation with CO_TD_ changes in PAH patients [13]. That technique was considered acceptable in most cases, despite LoA and PE similar to the present ones. Other promising techniques, such as cardiac magnetic resonance [17, 18] or echocardiography [19, 20], raise real interest in CO determination but require the advanced expertise of a dedicated technician and a specific setting.

We didn’t aim at showing whether uncorrected MF could be interchangeable with TD. Previous studies that addressed this question showed that this is not so [21]. Moreover, contrary to other clinical conditions, RHC is mandatory for PAH or CTEPH diagnosis [1]. Thus, since these patients undergo anyway RHC, it would be easy to obtain a correction factor [4, 22] and calibrate MF with basal TD in order to use the former for subsequent CO determination during various conditions.

In this study, we also showed that MF detects significant directional changes of CO associated to vasoreactivity testing, fluid challenge or exercise (Figs 4 and 5). Also transthoracic bioreactance was claimed to reliably detect dynamic, directional CO changes following vasoreactivity testing with a sensitivity and specificity of 88.9% and 100%. Our results are similar to those reported in this publication [14]. In a consistent manner with previous reports [23], we assumed that a CO_TD_ variation would be significant when ≥10%, although an actual significant variation should overtake the magnitude of the least significant change. Thus, the minimum change that a device must measure to detect a real change is given by the formula: precision multiplied by √2 [24]. From our data, the least significant change corresponded to a 9.2% change. When applying this limit for calculation of sensitivity and specificity, no value was reclassified.

The third finding of our study is that, once corrected against a reference method, MF showed acceptable accuracy and precision in determining CO changes (Figs 4 and 5). In fact, CO_MFcorr_ revealed LoA and PE below the limits for inter-changeability with TD. This suggests that CO_MFcorr_ may constitute an alternative method to TD for serial CO measurements on the same patient during the same procedure. The range of individual correction factors corresponds to that of healthy subjects [4]. So, MF application may simplify and shorten any RHC protocol where several CO measurements are required [1, 25]. Moreover, it makes possible to explore CO changes with a beat-by-beat resolution, allowing opportunities to describe more precisely cardiovascular adjustments during transients, like exercise onset in PH patients. In fact, there is a growing interest in exploring exercise haemodynamics in pulmonary vascular diseases. For instance, the establishment of multipoint pressure-flow (mPAP–CO) relationships during exercise was recently shown to better describe pulmonary vascular resistive properties than PVR calculated only from resting values [26, 27]. Our group also recently showed that exercise haemodynamics may also give invaluable information for unmasking pulmonary vascular disease [28], assessing PAH severity and driving its therapeutic approach [29]. Likewise, haemodynamic response to exercise may be useful in patients with high risk of developing PAH such as relatives of patients with heritable PAH [30] or patients with diseases associated with high risk of developing PAH or resting mPAP values between 20 and 25 mmHg [31]. Cardiodynamic exploration with determination of PVR changes at exercise may also help unmasking PH due to left heart disease that could mimic PAH at rest and induce misdiagnosing [32]. Finally, the most recent guidelines for PAH treatment strongly recommend the inclusion of patients in supervised exercise rehabilitation programmes [2], where the possibility of easy-access non-invasive monitoring of CO changes is desirable.

This study has several limitations. TD is not the gold standard for CO determination: the comparison with the direct Fick method may provide different results. Yet, TD reflects everyday clinical reality and common practice in PH reference centres and we aimed at comparing MF to routine procedure. MF is unusable in patients with poor finger pulse-wave signal, as it was the case in the present study for two patients with PAH associated to connective tissue diseases (CTD). This may be an issue in centers were PAH associated to CTD is the most represented population. It is also unsure if MF and TD correlation would be different in patients with cardiac arrhythmias, which was not the case for the patients included in our study. Finally, it is still uncertain that the validity of the individual correction factor determined during RHC persists over time or in different clinical testing. To date, individual correction of MF needs to be determined against a reference method at every new exploration. That said, our data still confirm that MF is reliable for relative CO changes even if no correction factor is applied. It is also noteworthy that the model of aortic compliance used to compute CO_MF_ was developed from data obtained on healthy subjects [3]. So it may be that the characteristics of aortic compliance in PAH or CTEPH patients are different, inducing here a potential bias. In our study, this issue was counterbalanced by the individual correction factors. Yet it may well impair the utilization of a population correction factor, which moreover would have been unwise to use in our study, considering the size of the study population and the variables to be included in the analysis. We acknowledge that this may be seen as a limitation of our study. Nevertheless, the mean correction factor determined in the present work was similar to those obtained previously in healthy subjects against another reference method [4].

## Conclusions

MF is a simple and reliable method for detecting CO changes in pre-capillary PH. Once corrected against a reference method, it is precise and accurate enough to evaluate absolute CO changes in response to exercise, fluid challenge or vasoreactivity testing. MF may simplify haemodynamic evaluation of pre-capillary PH in clinical practice and widen the range of follow-up and exploratory opportunities as it could potentially be used to study CO changes outside the RHC laboratory on a beat-by-beat basis.

## Supporting Information

S1 Table General Data. The table gives all the individual studied baseline parameters of the study population (n = 50) reported in the Fig 2, Tables 1–3.
(PDF)

S2 Table Data CO_MF_−CO_TD_. This table gives all the individual conditions and number of simultaneous CO_TD_ and CO_MF_ measurements reported in the Fig 3 and Table 4 (n = 98).
(PDF)

S3 Table York analysis. This table gives the calculated confidence intervals of linear regression obtained with York algorithm for intercept (*a* ± 1.96*SD) and slope (*b* ± 1.96*SD), and confidence interval of CO_MF_ (Fig 3) or CO_MFcorr_. (Figs 4 and 5) against any CO_TD_.
(PDF)

S4 Table Data CO_MFcorr_.−CO_TD_. This table gives all the conditions and number of simultaneous CO_TD_ and CO_MFcorr_. measurements reported in the Fig 4 (n = 48).
(PDF)
