# Supplementary material for: Non-Invasive Determination of Cardiac Output in Pre-Capillary Pulmonary Hypertension
Source: PLoS One. 2015 Jul 30;10(7):e0134221. doi: 10.1371/journal.pone.0134221 (PMC4520479; doi:10.1371/journal.pone.0134221)
Supplement: S1 Table — The table gives all the individual studied baseline parameters of the study population (n = 50) reported in the Fig 2, Tables 1–3. (PDF) [file pone.0134221.s001.pdf]

General data (n=50)

PAH

Patient n°	Age	Sex	Height	Weight	BMI	BSA	SAP	DAP	MAP	RAP	mPAP	dPAP	sPAP	PAWP	PVR	SVR	COTD	SD	COMF	SD	Bos	Sild	Tada	Epo	Trep	CCB	Comb
4	43		162	90	34.3	1.94	115	50	74	8	55	32	83	10	7.30	10.70	6.2	0.6	7.1	0.4	1	1		1			3
9	72		164	75	27.9	1.82	139	69	91	9	63	34	115	10	7.40	11.44	7.2	0.2	8.4	0.9	1						
10	54	1	165	85	31.2	1.92	122	61	83	9	43	20	71	12	4.56	10.88	6.8	0.1	6.5	0.4	1	1		1			3
14	57		160	60	23.4	1.62	154	68	94	1	36	15	60	7	5.61	18.00	5.2	0.4	6.4	0.6	1	1					2
28	29		168	68	24.1	1.77	123	61	84	7	62	47	89	10	10.00	14.81	5.2	0.9	5.3	0.7	1			1			2
31	78		159	82	32.4	1.84	111	86	103	9	51	10	27	10	9.11	20.89	4.5	0.0	5.0	0.6	1	1					2
33	38	1	164	63	23.4	1.69	124	75	93	5	35	20	58	8	5.23	17.05	5.2	0.2	6.4	1.0	1	1					2
34	25		152	67	29.0	1.64	131	70	101	2	49	36	75	11	6.71	17.47	5.7	2.3	6.2	0.3	1	1		1			3
45	62		155	68	28.3	1.67	137	64	89	0	33	20	55	5	5.38	17.12	5.2	0.1	7.2	0.4	1						
46	57	1	170	54	18.7	1.62	108	63	79	5	45	29	70	8	10.00	20.00	3.7	0.1	6.3	1.2	1	1					2
6	31	1	165	60	22.0	1.66	106	64	77	4	56	39	82	10	7.36	11.68	6.3	0.1	6.9	0.3	1	1		1			3
5	44	1	182	91	27.5	2.13	142	82	105	5	45	25	75	8	5.14	13.89	7.2	0.2	6.5	0.5					1		
38	55		160	63	24.6	1.66	144	63	84	4	60	43	82	8	7.09	10.91	7.3	0.3	6.3	0.7		1					
36	46		162	59	22.5	1.63	137	52	78	8	43	30	71	7	5.87	11.41	6.1	0.2	7.3	1.1						1	
27	47	1	175	53	17.3	1.64	82	64	69	12	48	30	86	7	9.46	13.15	4.3	0.3	3.8	0.2	1						
39	50		160	43	16.8	1.41	130	80	98	4	39	26	70	7	5.29	15.54	6.1	0.2	6.0	0.9	1						
41	27	1	183	80	23.9	2.02	138	78	96	7	44	30	64	9	2.98	7.59	11.7	0.2	9.9	0.6		1					
23	35	1	178	66	20.8	1.82	121	75	93	10	80	55	116	11	9.41	11.32	7.3	0.2	7.1	0.4	1		1				2
32	40	1	179	62	19.4	1.78	104	56	68	4	51	31	81	6	7.31	10.39	6.2	1.0	7.2	0.4	1			1			2
47	43	1	183	96	28.7	2.18	168	88	126	3	33	20	57	12	1.85	10.85	11.3	0.9	14.2	0.6		1					
65	40		165	59	21.7	1.65	143	70	112	3	47	31	59	10	8.22	24.22	4.5	0.1	6.8	1.1	1						
67	51	1	160	51	19.9	1.51	187	89	118	4	31	19	48	9	4.78	24.78	4.6	0.3	4.8	0.3							
19	72		166	107	38.8	2.13	171	69	98	8	30	16	41	13	1.92	10.17	8.9	0.1	9.6	1.0							
12	39	1	184	81	23.9	2.04	132	74	98	9	48	29	66	15	5.89	15.89	5.6	0.1	6.8	0.5							
24	65		161	51	19.7	1.52	124	75	93	7	35	23	61	2	12.89	33.59	2.6	0.3	4.1	0.3							
25	14		160	70	27.3	1.73	69	61	66	6	100	78	144	22	26.44	20.34	3.0	0.4	3.8	0.3							
30	53		165	60	22.0	1.66	109	82	93	0	41	28	62	2	11.25	26.83	3.5	0.1	3.3	0.4							
37	62		169	75	26.3	1.86	144	78	96	4	40	30	76	9	5.89	17.47	5.3	0.2	6.1	0.3							
42	58		158	62	24.8	1.63	126	71	90	6	63	40	98	9	6.00	9.33	9.0	0.6	10.1	0.8							
50	73		161	76	29.3	1.80	115	58	77	11	52	33	86	8	9.17	13.75	4.8	0.1	5.1	1.6							
**Total**	**48.7**	**12**	**166.5**	**69.2**	**25.0**	**1.77**	**128.5**	**69.9**	**90.9**	**5.8**	**48.6**	**30.6**	**74.3**	**9.2**	**7.5**	**14.7**	**6.0**	**0.3**	**6.7**	**0.6**	**16**	**11**	**1**	**6**	**1**	**1**	**11**
	**15.6**		**8.9**	**14.8**	**5.1**	**0.19**	**24.9**	**10.1**	**14.2**	**3.1**	**14.9**	**13.2**	**23.1**	**3.7**	**4.4**	**7.4**	**2.1**	**0.5**	**2.2**	**0.3**	**72.7**	**50.0**	**4.5**	**27.3**	**4.5**	**4.5**	**50.0**
	**%**	**60.0**																									

General data (n=50)

CTEPH

Patient n°	Age	Sexe	Height	Weight	BMI	BSA	SAP	DAP	MAP	RAP	mPAP	dPAP	sPAP	PAWP	PVRtd	SVR	TDCO	TDsd	MFCO	MFsd	Bos	Sild	Tada	Epo	Trep	CCB	Comb
3	56	1	174	73	24.1	1.87	139	77	95	11	45	26	80	5	8.76	18.39	4.6	0.3	4.1	0.3	1						
15	79		155	58	24.1	1.56	180	80	120	6	52	28	91	7	16.27	41.20	2.8	0.2	2.9	0.8							
18	65		172	80	27.0	1.93	171	93	133	4	33	20	53	6	5.91	28.25	4.6	0.2	4.8	0.5							
20	66		152	84	36.4	1.80	128	66	85	14	54	30	95	7	12.70	19.19	3.7	0.2	5.4	0.6							
21	64	1	171	70	23.9	1.82	146	77	99	5	60	35	111	8	10.99	19.86	4.7	0.2	4.8	0.1							
22	63		163	55	20.7	1.58	154	87	123	5	60	34	104	7	18.28	40.69	2.9	0.1	4.1	0.3							
29	36	1	190	83	23.0	2.11	128	74	90	3	30	19	47	6	3.19	11.55	7.5	0.5	7.1	0.3							
43	69		160	62	24.2	1.64	157	75	109	18	50	28	88	6	12.22	25.28	3.6	0.2	5.6	0.3							
44	68		164	70	26.0	1.76	179	84	122	6	42	25	75	10	10.91	39.55	2.9	0.3	5	1.3							
49	66	1	178	106	33.5	2.23	138	78	101	14	48	25	79	8	6.35	13.81	6.3	0.1	6.7	0.2							
53	53		155	50	20.8	1.47	154	106	134	0	11	1	21	1	2.29	30.69	4.4	0.3	4.8	0.5							
64	70	1	169	72	25.2	1.82	148	88	105	2	40	23	68	2	9.34	25.33	4.1	0.2	4.6	0.4							
26	64		152	66	28.6	1.63	95	59	74	5	39	19	69	8	6.89	15.33	4.5	0.2	5.6	0.5	1	1					2
52	81	1	162	46	17.5	1.46	96	53	70	10	38	18	74	6	9.14	17.14	3.5	0.1	3.1	0.8	1	1					2
**Total**	**64.3**	**6**	**165.5**	**69.6**	**25.4**	**1.76**	**143.8**	**78.4**	**104.3**	**7.4**	**43.0**	**23.6**	**75.4**	**6.2**	**9.5**	**24.7**	**4.3**	**0.2**	**4.9**	**0.5**	**3**	**2**	**0**	**0**	**0**	**0**	**2**
	**11.0**		**10.9**	**15.7**	**4.9**	**0.23**	**26.2**	**13.5**	**20.4**	**5.2**	**13.0**	**8.4**	**23.7**	**2.4**	**4.5**	**10.1**	**1.3**	**0.1**	**1.2**	**0.3**	**21.4**	**14.3**	**0.0**	**0.0**	**0.0**	**0.0**	**14.3**
	**%**	**57.1**																									
61	34		160	64	25.0	1.67	123	79	96	1	31	17	45	7	5.22	20.65	4.6	0.1	5.1	0.3	1	1					2
62	73	1	176	98	31.6	2.14	112	66	84	12	36	24	52	15	3.21	11.02	6.5	0.2	5.9	0.8	1		1				2
8	77	1	163	78	29.4	1.84	194	94	127	2	27	10	47	8	3.80	25.00	5.0	0.6	4.8	1.1							
16	35		169	97	34.0	2.07	116	63	76	4	15	8	23	6	1.54	12.34	5.8	0.2	7.8	0.8							
35	71		150	93	41.3	1.86	156	72	92	4	20	10	31	9	2.08	16.60	5.3	0.3	5.6	1.7							
57	47		162	72	27.4	1.77	141	74	94	3	29	17	43	11	3.12	15.78	5.8	0.2	8.0	0.7							
**Total**	**56.2**	**2**	**163.3**	**83.7**	**31.5**	**1.89**	**140.2**	**74.8**	**95.0**	**4.3**	**26.3**	**14.3**	**40.2**	**9.3**	**3.2**	**17.1**	**5.5**	**0.2**	**6.2**	**0.9**	**2**	**1**	**1**	**0**	**0**	**0**	**2**
	**19.8**		**8.8**	**14.3**	**5.8**	**0.18**	**34.7**	**12.4**	**19.5**	**3.9**	**7.6**	**6.1**	**10.9**	**3.3**	**1.3**	**5.8**	**0.7**	**0.2**	**1.4**	**0.5**	**33.3**	**16.7**	**16.7**	**0.0**	**0.0**	**0.0**	**33.3**
	**%**	**66.7**																									
