# Supplementary material for: Non-Invasive Determination of Cardiac Output in Pre-Capillary Pulmonary Hypertension
Source: PLoS One. 2015 Jul 30;10(7):e0134221. doi: 10.1371/journal.pone.0134221 (PMC4520479; doi:10.1371/journal.pone.0134221)
Supplement: S2 Table — This table gives all the individual conditions and number of simultaneous COTD and COMF measurements reported in the Fig 3 and Table 4 (n = 98). (PDF) [file pone.0134221.s002.pdf]

Data CO_{MF} - CO_{TD} (n=98)

Patient n°	PAH	CTEPH	State	COTD	SD	COMF	SD	CO mean	CO diff
8		1	B	5.0	0.6	4.8	1.1	4.9	-0.2
16		1	B	5.8	0.2	7.8	0.8	6.8	2.0
35		1	B	5.3	0.3	5.6	1.7	5.5	0.3
57		1	B	5.8	0.2	8.0	0.7	6.9	2.2
61		1	B	4.6	0.1	5.1	0.3	4.9	0.5
62		1	B	6.5	0.2	5.9	0.8	6.2	-0.6
3		1	B	4.6	0.3	4.1	0.3	4.3	-0.5
15		1	B	2.8	0.2	5.6	0.8	4.2	2.8
18		1	B	4.6	0.2	4.8	0.5	4.7	0.2
20		1	B	3.7	0.2	5.4	0.6	4.6	1.7
21		1	B	4.7	0.2	4.8	0.1	4.8	0.1
22		1	B	2.9	0.1	4.1	0.3	3.5	1.2
26		1	B	4.5	0.2	5.6	0.5	5.1	1.1
29		1	B	7.5	0.5	7.1	0.3	7.3	-0.4
43		1	B	3.6	0.2	5.6	0.3	4.6	2.0
44		1	B	2.9	0.3	5	1.3	4.0	2.1
49		1	B	6.3	0.1	6.7	0.2	6.5	0.4
52		1	B	3.5	0.1	3.1	0.8	3.3	-0.4
53		1	B	4.4	0.3	4.8	0.5	4.6	0.4
64		1	B	4.1	0.2	4.6	0.4	4.3	0.5
16		1	E	7.1	0.4	8.2	1.1	7.7	1.1
16		1	E	7.4	0.1	10.3	1.0	8.9	2.9
16		1	E	8.4	0.4	12.8	1.2	10.6	4.4
16		1	E	10.9	0.4	15.3	1.2	13.1	4.4
53		1	E	5.1	0.2	4.6	0.2	4.9	-0.5
53		1	E	6.3	0.4	6.0	0.4	6.2	-0.3
53		1	E	7.3	0.1	7.1	0.5	7.2	-0.2
29		1	F	7.2	0.2	7.2	0.5	7.2	0.0
3		1	NO	4.3	0.1	4.3	0.3	4.3	0.0
15		1	NO	3.2	0.2	5.8	0.7	4.5	2.6
18		1	NO	5.0	0.0	5.7	0.1	5.4	0.7
20		1	NO	3.5	0.2	5.8	0.8	4.7	2.3
21		1	NO	4.2	0.1	5.3	0.3	4.8	1.1
43		1	NO	3.4	0.1	5.3	0.1	4.4	1.9
44		1	NO	2.9	0.2	5.4	1.4	4.2	2.5
49		1	NO	6.2	0.4	7.2	0.3	6.7	1.0
64		1	NO	4.2	0.2	4.9	0.6	4.6	0.7
16		1	P	5.2	0.1	7.1	0.7	6.2	1.9
57		1	P	6.4	0.1	8.3	0.4	7.4	1.9
61		1	P	4.7	0.1	5.5	0.2	5.1	0.8
49		1	P	6.5	0.5	7.1	0.6	6.8	0.6
53		1	P	4.6	0.2	4.7	0.4	4.7	0.1
64		1	P	4.5	0.5	5.1	1.6	4.8	0.6
4	1		B	6.2	0.6	7.1	0.4	6.6	0.9
5	1		B	7.2	0.2	6.5	0.5	6.9	-0.7
6	1		B	6.3	0.1	6.9	0.3	6.6	0.7
9	1		B	7.2	0.2	8.4	0.9	7.8	1.2
10	1		B	6.8	0.1	6.5	0.4	6.7	-0.3
12	1		B	5.6	0.1	6.8	0.5	6.2	1.2
14	1		B	5.2	0.4	6.4	0.6	5.8	1.2
19	1		B	8.9	0.1	9.6	1.0	9.2	0.7
23	1		B	7.3	0.2	7.1	0.4	7.2	-0.2
24	1		B	2.6	0.3	4.1	0.3	3.3	1.5
25	1		B	3.0	0.4	3.8	0.3	3.4	0.9
27	1		B	4.3	0.3	3.8	0.2	4.1	-0.5
28	1		B	5.2	0.9	5.3	0.7	5.3	0.1
30	1		B	3.5	0.1	3.3	0.4	3.4	-0.2
31	1		B	4.5	0.0	5.0	0.6	4.8	0.5
32	1		B	6.2	1.0	7.2	0.4	6.7	1.0
33	1		B	5.2	0.2	6.4	1.0	5.8	1.2
34	1		B	5.7	2.3	6.2	0.3	5.9	0.5
36	1		B	6.1	0.2	7.3	1.1	6.7	1.2

Data CO_{MF} - CO_{TD} (n=98)

37	1	B	5.3	0.2	6.1	0.3	5.7	0.8
38	1	B	7.3	0.3	6.3	0.7	6.8	-1.0
39	1	B	6.1	0.2	6.0	0.9	6.0	-0.1
41	1	B	11.7	0.2	9.9	0.6	10.8	-1.8
42	1	B	9.0	0.6	10.1	0.8	9.6	1.1
45	1	B	5.2	0.1	7.2	0.4	6.2	2.0
46	1	B	3.7	0.1	6.3	1.2	5.0	2.6
47	1	B	11.3	0.9	14.2	0.6	12.8	2.9
50	1	B	4.8	0.1	5.1	1.6	5.0	0.3
65	1	B	4.5	0.1	6.8	1.1	5.7	2.3
67	1	B	4.6	0.3	4.8	0.3	4.7	0.2
36	1	E	7.9	0.5	9.9	0.7	8.9	2.0
36	1	E	8.9	0.4	10.1	0.6	9.5	1.2
36	1	E	9.6	0.0	11.2	0.7	10.4	1.6
36	1	E	9.9	0.2	11.9	0.6	10.9	2.0
46	1	E	5.1	0.1	7.4	0.7	6.3	2.3
47	1	E	10.9	0.4	14.5	0.7	12.7	3.6
47	1	E	12.7	0.2	15.2	0.6	14.0	2.5
50	1	E	5.4	0.0	5.5	1.8	5.5	0.1
50	1	E	5.7	0.4	6.2	2.1	6.0	0.5
19	1	F	10.0	0.7	10.0	0.6	10.0	0.0
24	1	F	2.7	0.1	3.6	0.2	3.2	0.9
30	1	F	3.5	0.3	3.7	0.4	3.6	0.2
31	1	F	4.7	0.1	5.9	0.6	5.3	1.2
12	1	NO	6.8	0.1	7.3	0.8	7.1	0.5
19	1	NO	9.4	0.4	10.2	0.6	9.8	0.8
24	1	NO	2.6	0.1	3.6	0.2	3.1	1.0
31	1	NO	5.0	0.1	6.1	0.4	5.6	1.1
36	1	NO	6.6	0.1	8.0	0.3	7.3	1.4
37	1	NO	6.0	0.1	7.7	0.4	6.9	1.7
50	1	NO	4.5	0.2	5.6	2.0	5.1	1.1
46	1	P	3.9	0.4	8.3	1.8	6.1	4.4
47	1	P	10.0	0.1	13.4	0.5	11.7	3.4
50	1	P	5.1	0.0	5.3	2.0	5.2	0.2
65	1	P	4.7	0.1	7.4	0.8	6.1	2.7
67	1	P	5.6	0.3	5.6	0.4	5.6	0.0
