# Supplementary material for: Non-Invasive Determination of Cardiac Output in Pre-Capillary Pulmonary Hypertension
Source: PLoS One. 2015 Jul 30;10(7):e0134221. doi: 10.1371/journal.pone.0134221 (PMC4520479; doi:10.1371/journal.pone.0134221)
Supplement: S3 Table — This table gives the calculated confidence intervals of linear regression obtained with York algorithm for intercept (a ± 1.96*SD) and slope (b ± 1.96*SD), and confidence interval of COMF (Fig 3) or COMFcorr. (Figs 4 and 5) against any COTD. (PDF) [file pone.0134221.s003.pdf]

York analysis

Figure 3A

CO	minus	max	mean
0	-0.060	1.092	0.516
1	0.938	2.258	1.598
2	1.936	3.424	2.680
3	2.934	4.590	3.762
4	3.932	5.756	4.844
5	4.930	6.922	5.926
6	5.928	8.088	7.008
7	6.926	9.254	8.090
8	7.924	10.420	9.172
9	8.922	11.586	10.254
10	9.920	12.752	11.336
11	10.918	13.918	12.418
12	11.916	15.084	13.500
13	12.914	16.250	14.582
14	13.912	17.416	15.664
15	14.910	18.582	16.746
16	15.908	19.748	17.828

Figure 3B

CO	minus	max	mean
0	-0.452	0.866	0.207
1	0.593	2.155	1.374
2	1.638	3.444	2.541
3	2.683	4.733	3.708
4	3.728	6.022	4.875
5	4.773	7.311	6.042
6	5.818	8.600	7.209
7	6.863	9.889	8.376
8	7.908	11.178	9.543
9	8.953	12.467	10.710
10	9.998	13.756	11.877
11	11.043	15.045	13.044
12	12.088	16.334	14.211
13	13.133	17.623	15.378
14	14.178	18.912	16.545
15	15.223	20.201	17.712
16	16.268	21.490	18.879

Figure 3C

CO	minus	max	mean
0	0.083	0.902	0.493
1	1.111	2.064	1.588
2	2.139	3.226	2.683
3	3.167	4.388	3.778
4	4.195	5.550	4.873
5	5.223	6.712	5.968
6	6.251	7.874	7.063
7	7.279	9.036	8.158
8	8.307	10.198	9.253
9	9.335	11.360	10.348
10	10.363	12.522	11.443
11	11.391	13.684	12.538
12	12.419	14.846	13.633
13	13.447	16.008	14.728
14	14.475	17.170	15.823
15	15.503	18.332	16.918
16	16.531	19.494	18.013

Figur 4A

CO	minus	max	mean
0	-0.404	0.591	0.094
1	0.512	1.655	1.084
2	1.428	2.719	2.074
3	2.344	3.783	3.064
4	3.260	4.847	4.054
5	4.176	5.911	5.044
6	5.092	6.975	6.034
7	6.008	8.039	7.024
8	6.924	9.103	8.014
9	7.840	10.167	9.004
10	8.756	11.231	9.994
11	9.672	12.295	10.984
12	10.588	13.359	11.974
13	11.504	14.423	12.964
14	12.420	15.487	13.954
15	13.336	16.551	14.944
16	14.252	17.615	15.934

Figure 5A

CO	minus	max	mean
0	-2.583	0.397	-1.093
1	-1.644	1.692	0.024
2	-0.705	2.987	1.141
3	0.234	4.282	2.258
4	1.173	5.577	3.375
5	2.112	6.872	4.492
6	3.051	8.167	5.609
7	3.990	9.462	6.726
8	4.929	10.757	7.843
9	5.868	12.052	8.960
10	6.807	13.347	10.077
11	7.746	14.642	11.194
12	8.685	15.937	12.311
13	9.624	17.232	13.428
14	10.563	18.527	14.545
15	11.502	19.822	15.662
16	12.441	21.117	16.779

	intercept (a)	slope (b)
Figure 3A	a=0.207+/-0.336	b=1.167+/-0.062
Figure 3B	a=0.516+/-0.294	b=1.082+/-0.043
Figure 3C	a=0.493+/-0.209	b=1.095+/-0.034
Figure 4A	a=0.094+/-0.254	b=0.990+/-0.038
Figure 5A	a=-1.093+/-0.760	b=1.117+/-0.091
