# Supplementary material for: Non-Invasive Determination of Cardiac Output in Pre-Capillary Pulmonary Hypertension
Source: PLoS One. 2015 Jul 30;10(7):e0134221. doi: 10.1371/journal.pone.0134221 (PMC4520479; doi:10.1371/journal.pone.0134221)
Supplement: S4 Table — This table gives all the conditions and number of simultaneous COTD and COMFcorr. measurements reported in the Fig 4 (n = 48). (PDF) [file pone.0134221.s004.pdf]

Data $CO_{MFcorr.}$ - CO_{TD} (n=48)

Patient n°	State	COMFcorr.	SD	COTD	SD	Mean	Diff.
3	NO	4.8	0.3	4.3	0.1	4.5	0.5
12	NO	6.0	0.7	6.8	0.1	6.4	-0.8
15	NO	2.9	0.3	3.2	0.2	3.1	-0.3
16	E	6.1	0.8	7.1	0.4	6.6	-1.0
16	E	7.7	0.7	7.4	0.1	7.6	0.3
16	E	9.6	0.9	8.4	0.4	9.0	1.2
16	E	11.4	0.9	10.9	0.4	11.2	0.5
16	P	5.3	0.5	5.2	0.1	5.3	0.1
18	NO	5.4	0.1	5.0	0.0	5.2	0.4
19	F	9.2	0.6	10.0	0.7	9.6	-0.8
19	NO	9.4	0.6	9.4	0.4	9.4	0.0
20	NO	4.0	0.5	3.5	0.2	3.7	0.5
21	NO	5.2	0.3	4.2	0.1	4.7	1.0
24	F	2.2	0.1	2.7	0.1	2.5	-0.5
24	NO	2.2	0.1	2.6	0.1	2.4	-0.4
29	F	7.6	0.5	7.2	0.2	7.4	0.4
30	F	3.9	0.4	3.5	0.1	3.7	0.4
31	F	5.3	0.5	4.7	0.1	5.0	0.6
31	NO	5.5	0.4	5.0	0.1	5.2	0.5
36	E	8.3	0.6	7.9	0.5	8.1	0.4
36	E	8.5	0.5	8.9	0.4	8.7	-0.4
36	E	9.4	0.6	9.6	0.0	9.5	-0.2
36	E	10.0	0.5	9.9	0.2	9.9	0.1
36	NO	6.7	0.3	6.6	0.1	6.7	0.1
37	NO	6.6	0.3	6.0	0.1	6.3	0.6
43	NO	3.4	0.1	3.4	0.1	3.4	0.0
44	NO	3.2	0.8	2.9	0.2	3.0	0.3
46	E	4.3	0.4	5.1	0.1	4.7	-0.8
46	P	4.9	1.1	3.9	0.4	4.4	1.0
47	E	11.6	0.6	10.9	0.4	11.2	0.7
47	E	12.1	0.5	12.7	0.2	12.4	-0.6
47	P	10.7	0.4	10.0	0.1	10.3	0.7
49	NO	6.8	0.3	6.2	0.4	6.5	0.6
49	P	6.7	0.6	6.5	0.5	6.6	0.2
50	E	5.2	1.7	5.4	0.0	5.3	-0.2
50	E	5.8	2.0	5.7	0.4	5.8	0.1
50	NO	5.3	1.9	4.5	0.2	4.9	0.8
50	P	5.0	1.9	5.1	0.0	5.0	-0.1
53	E	4.2	0.2	5.1	0.2	4.6	-0.9
53	E	5.5	0.4	6.3	0.4	5.9	-0.8
53	E	6.5	0.5	7.3	0.1	6.9	-0.8
53	P	4.3	0.4	4.6	0.2	4.4	-0.3
57	P	4.8	0.3	6.4	0.1	5.6	-1.6
61	P	4.8	0.2	4.7	0.1	4.8	0.1
64	NO	4.8	0.5	4.2	0.2	4.5	0.6
64	P	4.5	1.4	4.5	0.1	4.5	0.0
65	P	4.8	0.5	4.7	0.1	4.8	0.1
67	P	4.8	0.4	5.6	0.3	5.2	-0.8
